# Identification of loci associated with susceptibility to bovine paratuberculosis and with the dysregulation of the *MECOM*, *eEF1A2*, and U1 spliceosomal RNA expression

**DOI:** 10.1038/s41598-020-79619-x

**Published:** 2021-01-11

**Authors:** Maria Canive, Nora Fernandez-Jimenez, Rosa Casais, Patricia Vázquez, José Luis Lavín, José Ramón Bilbao, Cristina Blanco-Vázquez, Joseba M. Garrido, Ramón A. Juste, Marta Alonso-Hearn

**Affiliations:** 1 Department of Animal Health, NEIKER- Basque Research and Technology Alliance (BRTA), Derio, Bizkaia Spain; 2 Doctoral Program in Immunology, Microbiology and Parasitology, Universidad del País Vasco/Euskal Herriko Unibertsitatea (UPV/EHU), Leioa, Bizkaia Spain; 3 Department of Genetics, Physical Anthropology and Animal Physiology, University of the Basque Country (UPV/EHU), Biocruces-Bizkaia HRI, Leioa, Bizkaia Spain; 4 SERIDA, Servicio Regional de Investigación y Desarrollo Agroalimentario, Grupo NySA, Deva, Asturias Spain; 5 SALUVET-Innova S.L., Faculty of Veterinary Sciences, Complutense University of Madrid, Madrid, Spain; 6 Department of Applied Mathematics, NEIKER-Basque Research and Technology Alliance (BRTA), Derio, Bizkaia Spain

**Keywords:** Immunology, Microbiology

## Abstract

Although genome-wide association studies have identified single nucleotide polymorphisms (SNPs) associated with the susceptibility to *Mycobacterium avium* subsp. *paratuberculosis* (*MAP*) infection, only a few functional mutations for bovine paratuberculosis (PTB) have been characterized. Expression quantitative trait loci (eQTLs) are genetic variants typically located in gene regulatory regions that alter gene expression in an allele-specific manner. eQTLs can be considered as functional links between genomic variants, gene expression, and ultimately phenotype. In the current study, peripheral blood (PB) and ileocecal valve (ICV) gene expression was quantified by RNA-Seq from fourteen Holstein cattle with no lesions and with PTB-associated histopathological lesions in gut tissues. Genotypes were generated from the Illumina LD EuroG10K BeadChip. The associations between gene expression levels (normalized read counts) and genetic variants were analyzed by a linear regression analysis using *R Matrix eQTL 2.2.* This approach allowed the identification of 192 and 48 *cis*-eQTLs associated with the expression of 145 and 43 genes in the PB and ICV samples, respectively. To investigate potential relationships between these *cis*-eQTLs and MAP infection, a case–control study was performed using the genotypes for all the identified *cis*-eQTLs and phenotypical data (histopathology, ELISA for MAP-antibodies detection, tissue PCR, and bacteriological culture) of 986 culled cows. Our results suggested that the heterozygous genotype in the *cis*-eQTL-rs43744169 (T/C) was associated with the up-regulation of the MDS1 and EVI1 complex (*MECOM*) expression, with positive ELISA, PCR, and bacteriological culture results, and with increased risk of progression to clinical PTB. As supporting evidence, the presence of the minor allele was associated with higher *MECOM* levels in plasma samples from infected cows and with increased MAP survival in an ex-vivo macrophage killing assay. Moreover, the presence of the two minor alleles in the *cis*-eQTL-rs110345285 (C/C) was associated with the dysregulation of the eukaryotic elongation factor 1-α2 (*eEF1A2*) expression and with increased ELISA (OD) values. Finally, the presence of the minor allele in the *cis*-eQTL rs109859270 (C/T) was associated with the up-regulation of the U1 spliceosomal RNA expression and with an increased risk of progression to clinical PTB. The introduction of these novel functional variants into marker-assisted breeding programs is expected to have a relevant effect on PTB control.

## Introduction

Paratuberculosis (PTB) is a gastrointestinal disease of domestic and wild ruminants caused by *Mycobacterium avium* susbp. *paratuberculosis* (MAP). PTB causes direct economic losses to the dairy farms due to decreased milk production, replacement cost, and premature culling or death from the clinical disease^1,2^. The most common clinical signs are progressive weight loss, diarrhea, and decreased milk yield^3^. PTB is a major problem for animal health and must be notified to the World Organization for Animal Health. In addition, MAP is a suspected cause of Crohn’s disease (CD) in humans^4^. Colorectal cancer is a complication of the two forms of idiopathic inflammatory bowel disease (IBD); colonic CD and ulcerative colitis. Interestingly, MAP bacilli have been detected in the intestines of patients with CD, ulcerative colitis, and IBD-associated colorectal cancer^5,6^.

Improving host genetics through selective breeding could enhance natural resistance to MAP infection and complement existing control strategies^7–9^. Over the last decade, different strategies including case–control studies in candidate genes and genome-wide association studies (GWAS) identified single nucleotide polymorphisms (SNPs) significantly associated with susceptibility to bovine PTB^10–16^. However, few SNPs were identified with consistent association across studies and the molecular mechanisms by which they contribute to disease remain largely unknown. Recent studies using RNA next-generation sequence technology (RNA-Seq) have been conducted to gain a better understanding of the molecular mechanisms involved in the circulating and local response against a natural MAP infection^17–19^. While transcriptomic studies provide insight into the genes and mechanisms involved in the response to MAP infection, they do not reveal whether there is variation in the transcriptional response among individuals and how this might influence PTB outcome.

**Figure 1:**
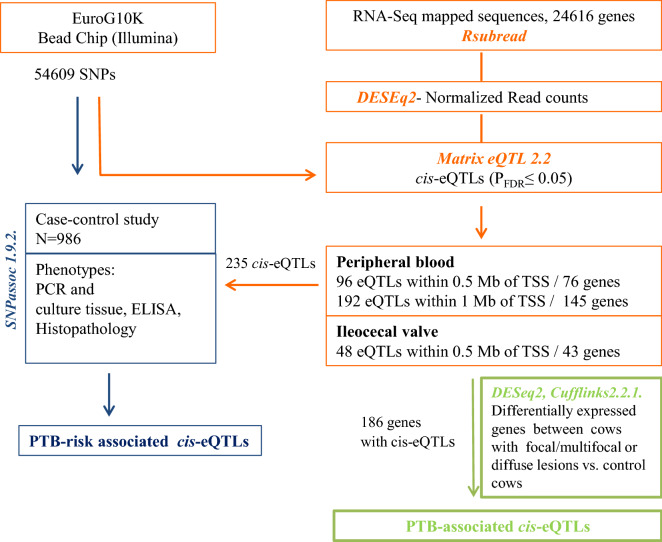
Study design. The approach starts from the individual expression levels, then identifies genetic variants controlling differences in gene expression, and finally checks that the identified *cis*-eQTLs are indeed associated with disease outcome.

Jansen and Nap^20^ introduced the concept of “genetical genomics” where genomic variants were associated with transcript abundance which acts as a heritable endophenotype. This concept supported the idea that integrating genomic variants and gene expression data could contribute to a better understanding of the genetic architecture underlying disease outcome (an endpoint phenotype)^21,22^. Polymorphisms associated with gene expression are known as expression quantitative trait loci (eQTLs). In a *cis* position (within 1 Mb upstream of the transcription start site of a gene locus), *cis*-eQTLs might alter transcription regulation, mRNA stability, or mRNA splicing^23,24^. This leads to changes in the level, timing, and/or localization of gene expression which can significantly influence variations in individual phenotype. In the current study, we hypothesized that individual genetic variation in the transcriptional response to PTB infection might have implications in disease outcome. The main objective was to identify *cis*-eQTLs significantly associated with changes in gene expression levels in peripheral blood (PB) and ileocecal valve (ICV) samples collected from Holstein cattle with no lesions and with PTB-associated lesions in gut tissues. Next, we assessed whether the genes with *cis*-eQTLs were DE in PTB-infected cows versus control cows. Finally, to test whether the identified *cis*-eQTLs affected not just gene expression but also the PTB risk, a case–control study using the genotypes of all the identified *cis*-eQTLs and the phenotypical data of a cohort of 986 culled cattle was performed. The workflow of the study is presented in Fig. 1.

## Results

### RNA-Seq data and genotypes

Samples from the animals included in this study were tested by ELISA for the detection of MAP-specific antibodies, qPCR for MAP DNA detection, bacteriological culture, and histopathological analysis, as previously described (Table 1)^18^. All control animals (N = 4) were negative for all the diagnostic tests. In contrast, the infected cows had detectable lesions in gut tissues with distinct severity; focal/multifocal (N = 6) or diffuse (N = 4). RNA-extraction from PB and ICV samples of the fourteen cows, RNA-Seq libraries preparation, and sequencing using an Illumina NextSeq500 sequencer were performed as previously described^18^. The raw reads were filtered by their length (minimum size 75 bp long) and percentage of ambiguous base N less than 10% using *Prinseq-lite 0.20.4*^25^. Then, the trimmed sequences were mapped to the *Bos Taurus* reference genome (Bos_taurus.UMD3.1. version 87) with *TopHat 2.1.1.*^26^*.* In the current study, the mapped reads were quantified with *RSubread* to calculate gene expression levels (read counts)^27^. In addition, genomic DNA was extracted from PB samples of the fourteen animals and subsequently genotyped using the Illumina MD EuroG10K BeadChip (54,609 SNPs) as previously described^28^. Altogether the study is comprised of 54,609 genetic variants and expression values of 24,616 genes in 28 samples.

**Table 1:** Histopathological analysis, ELISA, PCR, and bacteriological culture results from all the animals included in the current study.

Animal ID	Histopathological analysis			ELISA (OD)	Fecal PCR (DNA copies/g 10^2^)	Fecal culture	Tissue PCR (DNA copies/g 10^2^)	Tissue culture
	Macroscopic	Microscopic	ZN					
1	No	Negative	Negative	Negative (1.93)	Negative	Negative	Negative	Negative
2	No	Negative	Negative	Negative (8.54)	Negative	Negative	Negative	Negative
3	No	Negative	Negative	Negative (2.45)	Negative	Negative	Negative	Negative
15	No	Negative	Negative	Negative (1.72)	Negative	Negative	Negative	Negative
4	No	Focal	Negative	Negative (2.71)	Negative	Negative	Positive (0.38)	Negative
5	No	Focal	Negative	Negative (1.03)	Negative	Negative	Positive (7.50)	Low
6	No	Focal	Negative	Negative (5.51)	Negative	Negative	Positive (305.28)	Negative
7	No	Focal	Negative	Negative (0.74)	Negative	Negative	Positive (12.26)	Negative
8	No	Focal	Negative	Positive (133.17)	Positive	Negative	Positive (70.54)	Medium
9	No	Multifocal	Positive	Negative (2.97)	Negative	Negative	Negative	Negative
10	Yes	Diffuse	Positive	Positive (283.13)	Positive	Negative	Positive (667.65)	Heavy
11	Yes	Diffuse	Positive	Positive (187.69)	Positive (0.14)	Negative	Positive (3896.19)	Heavy
12	Yes	Diffuse	Positive	Positive (241.18)	Positive (114.00)	Heavy	Positive (104,032)	Heavy
14	Yes	Diffuse	Positive	Positive (255.55)	Positive (2832.00)	Negative	Positive (173,316.00)	Heavy

DNA samples with a PCR-positive result using the LSI VetMax Triplex real-time PCR were quantified using the ParaTB Kuanti-VK kit. qPCR results are expressed as MAP DNA copies per gram of feces or tissues × 10^2^. Bacterial load was classified as low (< 10 CFU), medium (between 10 to 50 CFU) or heavy (> 50 CFU).

*ZN* Ziehl–Neelsen, *OD* optical density, *CFU* colony forming units.

### *cis*-eQTL analysis

**Figure 2:**
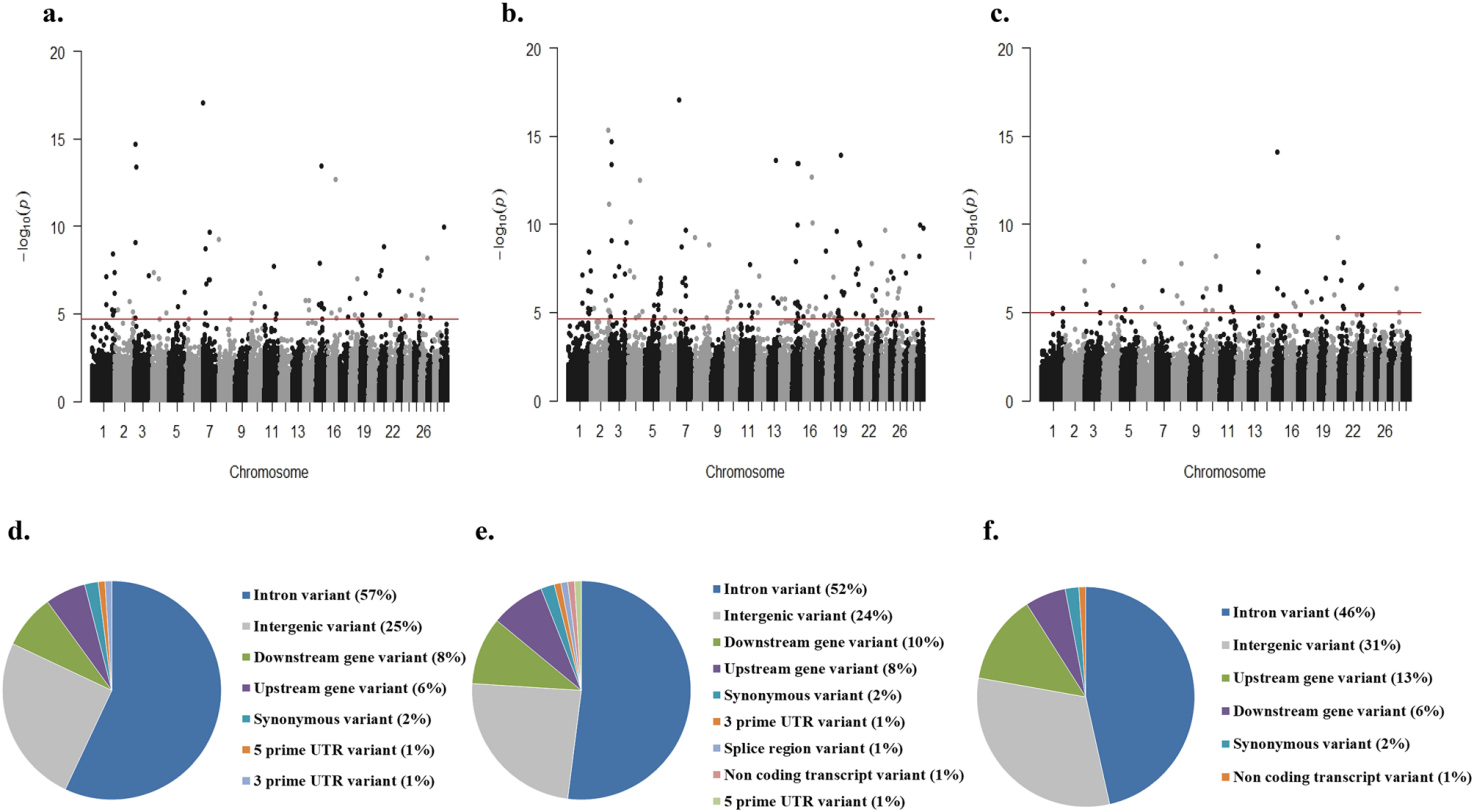
*cis*-eQTL analysis. (**a**,**b**) PB *cis*-eQTLs identified within 0.5 (**a**) and 1 Mb (**b**) upstream of a TSS. (**c**) ICV *cis*-eQTLs identified within 0.5 Mb upstream of a TSS. The plots show the − log_10_ (P-values) of the most significant variants for each of the 24,616 analyzed genes. The red line represents the threshold of P = 2.03E−05 (**a**), P = 2.14E-05 (**b**) and P = 1.02E−05 (**c**). (**d**,**e**) The chart depicts the genomic distribution of the PB-*cis*-eQTLs identified within 0.5 (**d**) and 1 Mb (**e**) upstream a TSS according to the Ensembl Variant Effect Predictor (VEP). (**f**) Genomic distribution of the ICV *cis*-eQTLs according to the VEP.

Gene expression data (read counts) were normalized with the mean-of-ratios method included in the *DESeq 2* package^29^. Our integrated genomic-transcriptomic dataset was analyzed for associations between SNPs-normalized read counts using *R Matrix eQTL 2.2*^30^ including the age of slaughter as a covariate. *Matrix eQTL* tests for associations between SNPs-normalized read counts by modeling the effect of genotype as additive linear (least square model). *cis*-eQTLs were identified by including all the variants on the same chromosome that are located 1 Mb upstream of the transcription start site (TSS) of a gene locus. A genetic variant was regarded as a significant *cis*-eQTL for a given gene if P_FDR_ ≤ 0.05. For the PB samples, two analyses were performed including all the tested genetic variants located within 0.5 and 1 Mb upstream of the TSS of a gene locus; Fig. 2a,b respectively. Using the 24,616 mapped genes, we identified 96 and 192 *cis*-eQTLs which were associated with 76 and 145 genes, respectively. As expected, all the cis-eQTLs identified in the first analysis were validated when the analysis was extended 1 Mb upstream of the TSS of a gene locus. Using the ICV datasets, the SNPs-reads association analysis allowed the identification of 48 *cis*-eQTLs located within 0.5 Mb upstream of a TSS and associated with the expression of 43 genes (Fig. 2c). As expected, all the identified *cis*-eQTLs were located on the same chromosome as the associated gene. Most of the identified *cis*-eQTLs were located in intronic or intergenic regions (Fig. 2d–f). The complete *cis*-eQTL discovery in the PB and ICV samples is presented in Tables S1–S3.

**Figure 3:**
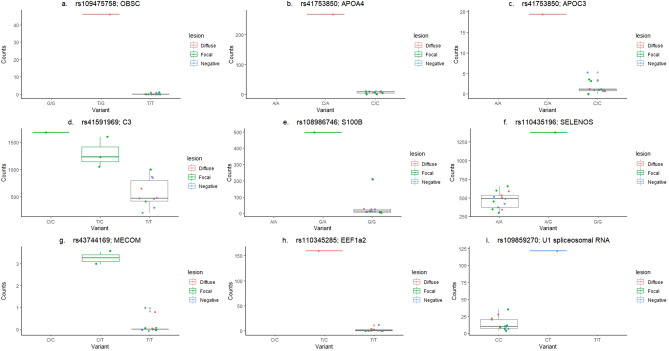
Boxplots for nine significant *cis*-eQTLs associations with gene expression. The rs109475758 (**a**), rs41753850 (**b**,**c**), rs41591969 (**d**), rs108986746 (**e**), rs110435196 (**f**), rs43744169 (**g**), rs110345285 (**h**) and rs109859270 (**i**) were associated with the *OBSC, APOA4, APOC3, C3, S100B, SELENOS*, *MECOM*, *eEF1A2* and U1 spliceosomal RNA expression, respectively. Gene variants are presented on the horizontal axis and gene expression levels, normalized gene counts, on the vertical axes. Samples were grouped according to the absence of lesions (negative) or the presence of focal/multifocal and diffuse lesions in gut tissues. The median and the upper and lower quartiles of each group are represented by horizontal and vertical lines, respectively. The lowest point is the minimum of the data set and the highest point is the maximum of the data set.

The *cis*-eQTL (rs109475758) with the lowest P_FDR_ (P_FDR_ = 4593E−14) in the PB dataset was associated with the *obscurin* (*OBSC*) gene (Table S1), a highly mutated gene in different types of cancer. The rs109475758 heterozygous genotype (T/G) resulted in increased counts of the *OBSC* gene while the most frequent homozygous genotype (T/T) correlated with low levels of *OBSC* gene expression (Fig. 3a). In the ICV samples, the gene with the strongest *cis*-eQTL (rs41753850) association was the *apolipoprotein A4* gene (*APOA4* ; P_FDR_ = 8413E−11) (Table S3). *APOA4* is associated with circulating high-density lipoproteins (HDL) and plays an important role in cholesterol transport and lipid metabolism. Interestingly, the rs41753850 was also associated with the *apolipoprotein C3* (*APOC3*) gene expression (P_FDR_ = 0.0031). *APOC3* plays an important role in regulating the metabolism of triglyceride-rich lipoproteins and has been regarded as a main component of chylomicrons and very-low-density lipoproteins (VLDL). The heterozygous genotype in the rs41753850 (C/A) resulted in increased levels of *APOA4* and *APOC3* expression*,* while the most frequent homozygous (C/C) correlated with lower levels of *APOA4* and *APOC3* gene expression (Fig. 3b,c)*.*

### Association between the genes with *cis*-eQTLs and MAP infection

We assessed whether the genes with *cis-* eQTLs were DE in PTB-infected cows versus controls (Table 2) using the differential expression information generated with the *Cufflinks* package *2.2.1.* in our previous study^18^ and with *DESeq2* in the current study. Despite the small number of control cows, we found that 24 of the genes with *cis*-eQTLs were DE in the PB samples from MAP-infected cows with focal/multifocal (N = 6) or diffuse lesions (N = 4) versus control cows (N = 4) (Table 2). Some of these *cis*-eQTLs-associated genes play key roles in tumorigenicity (*OBSC, GSTO1*), the apoptotic process (*CD5L, BNIP3*), transcription regulation (*PERM1*), cell adhesion (*NRCAM*), platelet aggregation (*GP1Bβ, MRVI1*), G-protein coupled receptor signaling pathway (*ADCY8, ACKR1*), lymphocyte activation (*HS1BP3*), adhesion of blood neutrophils in cytokine-activated endothelium (*Selectin E)* and in the regulation of hypoxia-induced autophagy (*ANKRD37)* in MAP-infected cattle. In addition, we found that fourteen genes with *cis*-eQTLs were DE in the ICV samples from MAP-infected cows with focal/multifocal or diffuse lesions versus control cows (Table 2). Some of these *cis*-eQTLs-associated genes play key roles in the extracellular matrix structure (*ACAN*), negative regulation of *NF-kβ* (*TRIM40*), innate immune response (*NEURL3, ARSB, Duosenase-1 like*), tumorigenicity (*PROM2*), G-protein coupled receptor signaling pathway (*ACKR1*), and oxidative stress (*PRDX1*) in MAP-infected cattle.

**Table 2:** Genes with cis-eQTLs that are differentially expressed in response to MAP infection.

Dataset	eQTL	Gene	*Cufflinks 2.2.1*-fold change (log_2_)		*DESeq2*-fold change (log_2_)	
			Focal vs control	Diffuse vs control	Focal vs control	Diffuse vs control
PB	rs109475758	*Obscurin (OBSC)*		5.6		
	rs29014816	*CD5 molecule like (CD5L)*			− 3.6	− 4.2
	rs109079658	*PPARGC1 and ESRR induced regulator (PERM1)*		4.7		
	rs109744678	*BCL2 interacting protein 3 (BNIP3)*				− 1.9
	rs41587635	*Neuronal cell adhesion molecule (NRCAM)*	1.7			
	rs110756953	*Glycoprotein Ib platelet subunit beta (GB1BB)*		− 1.8	− 1.7	
	rs109820008	*Adenylate cyclase 8 (ADCY8)*		1.7		
	rs109230217	*Desmin (DES)*		4.1		3.7
	rs108972532	*Desmin (DES)*		4.1		3.7
	rs110397138	*Murine retrovirus integration site 1 (MRVI1)*	− 1.4	− 1.8		
	rs41665220	Protein coding (ENSBTAG00000009359.6)		− 1.2		
	rs109535431	*Calpain 5 (CAPN5)*		− 1.2		− 2.0
	rs110203175	*Chimerin 1 (CHN1)*		− 2.5		
	rs41658581	*Calpain 2 (CAPN2)*		− 0.6		
	rs41591969	*Complement C3*	1.0		1.3	
	rs108986746	*S100 calcium binding protein B (S100B)*		− 2.1		
	rs110435196	*Selenoprotein S (SELENOS)*	− 0.8	− 0.7		
	rs42094373	*Glutathione S-transferase omega 1 (GSTO1)*				3.0
	rs110866743	*HCLS1 binding protein 3 (HS1BP3)*		− 0.9		
	rs41579631	*Selectin E (SELE)*		− 2.8		
	rs41636355	*Selectin E (SELE)*		− 2.8		
	rs41646333	*Epiregulin (EREG)*		− 4.8		
	rs110380638	*Ankyrin repeat domain 37 (ANKRD37)*	− 2.0	− 2.4		
	rs109003203	*N-acetylneuraminate pyruvate lyase (NPL)*	− 2.7			
ICV	rs41753850	*Apolipoprotein A* 4 (APOA4)		4.0		3.6
	rs109464338	*Kelch domain containing 7A* (KLHDC7A)	2.0			
	rs41621303	*Aggrecan* (ACAN)				− 2.5
	rs111006498	*Tripartite motif containing 40* (TRIM40)		3.0		
	rs29014822	*Neuralized E3 ubiquitin protein ligase 3* (NEURL3)				3.4
	rs41257569	*Arylsulfatase B* (ARSB)	− 0.8			
	rs41753850	*Apolipoprotein C3* (APOC3)		4.2		4.5
	rs43708883	*Actinin alpha 2* (ACTN2)		− 2.1		
	rs110406596	*Prominin 2* (PROM2)				− 2.5
	rs109082401	*TLC domain containing 2* (TLCD2)	− 2.0	− 2.8		
	rs109080794	*Metallothionein 1E* (MT1E)		3.8		2.6
	rs109502147	*Atypical chemokine receptor 1* (ACKR1)	− 0.8	− 1.2		− 2.0
	rs41976219	*Duodenase-1-like* (LOC786126)	− 3.6	− 2.0		
	rs42581502	*Peroxiredoxin 1* (PRDX1)		− 0.7		

To identify common biological functions, enrichment analysis of biological processes was performed with the *cluster Profiler Bioconductor package 3.10.1.*^31^ using all genes with *cis*-eQTLs. The rationale is that any change to a single gene involved in a process could alter many genes in a biological process and thus the phenotype of the animal. Gene ontology enrichment analysis indicated that three genes (*C3, S100B, SELENOS*) with *cis-* eQTLs were associated with the regulation of acute inflammatory response (GO:0002673) which was significantly enriched (P_FDR_ ≤ 0.05). The heterozygous genotype in the *cis*-eQTLs of the *C3* (Fig. 3d), *S100B* (Fig. 3e)*,* and *SELENOS* (Fig. 3f) genes resulted in higher gene expression levels; P_FDR_ = 0.0254, P_FDR_ = 0.0315 and P_FDR_ = 0.0320, respectively. In addition, the functional analysis revealed that the cholesterol metabolic route (bta04979) was significantly enriched, with the *APOA4* and *APOC3* genes involved in this route (P_FDR_ = 0.009, Gene ratio: 2/46). This finding reinforced the idea that the *APOA4-APOC3* gene cluster may be associated with the PTB risk.

### Case–control association study

If the expression levels of a *cis*-eQTL have any effect on the PTB risk, we should observe differences in disease phenotypes among the different genotypes. To address this hypothesis, a case–control association study using the genotypes for the 235 identified *cis*-eQTLs and phenotypical data of 986 cull Holstein Friesian cows was performed. The infection status of these cows was previously determined by histopathological analysis, ELISA, PCR, and bacteriological culture^32^. Cattle were classified as cases if they were positive for ELISA, PCR culture, and/or if they had PTB-associated lesions in gut tissues. The 986 cows were genotyped for the identified *cis*-eQTLs and cows with a genotyping rate < 80% were excluded. SNPs deviated significantly from the Hardy–Weinberg equilibrium were removed^33^. Case–control associations were analyzed with the WGassociation function of *SNPassoc 1.9.2* under five different genetic models (co-dominant, dominant, recessive, over-dominant, and log-additive)^34^. P-values were adjusted for multiple comparisons using the Bonferroni correction. As seen in Table 3, the rs43744169, rs110345285, and rs109859270 showed significant associations with PTB phenotypes. The association analysis using the *cis*-eQTLs identified with the PB dataset and a minor allele frequency (MAF) > 20% revealed significant associations between the *cis*-eQTL-rs43744169 (T/C) located on chromosome 1 and several disease phenotypes including histopathology, ELISA (OD), and ELISA, PCR, and bacteriological culture results. The heterozygous genotype (T/C) in the *cis*-eQTL-rs43744169 was associated with the up-regulation of the MDS1 and EVII complex (*MECOM*) expression (Fig. 3g; P_FDR_ = 0.00003).

**Table 3:** Associations between the rs43744169, rs110345285, rs109859270, and PTB diagnostic results.

Sample	eQTL	Phenotype	*SNPassoc1.9.2*-P values^a^				
			Codominant	Dominant	Recessive	Overdominant	Log-additive
PB	rs43744169 (T/C)	Histopathology^b^	**0.00011**	**0.00209**	**0.00028**	0.09028	**0.00011**
		ELISA (OD)	**0.00003**	**0.00059**	**0.00021**	0.04284	**0.00002**
		ELISA + PCR + Cultive	**0.00026**	**0.00012**	0.01932	0.00494	**0.00005**
	rs110345285 (T/C)	ELISA (OD)	**0.00007**	0.81100	**0.00001**	0.26584	0.16103
ICV	rs109859270 (C/T)	Histopathology^c^	**0.00226**	**0.00049**	0.45219	**0.00098**	**0.00112**

^a^Statistically significant P-values after Bonferroni correction are shown in bold.

^b^PTB-associated histopathological lesion were classified as focal, multifocal, diffuse paucibacillary, diffuse intermediate, diffuse multibacillary.

^c^Presence or absence of PTB-histopathological lesions.

Using a MAF > 10%, an association between the *cis*-eQTL-rs110345285 (T/C) and mean ELISA (OD) values was observed under the codominant and recessive genetic models. The presence of the two minor alleles in the *cis*-eQTL-rs110345285 (C/C) regulating the eukaryotic elongation factor 1-α2 (*eEF1A2)* expression was associated with a significant increase in mean OD values (OD = 0.80) when compared with cows with the most frequent homozygous genotype (T/T) (OD = 0.29) or with the heterozygous genotype (T/C) (OD = 0.26). The heterozygous genotype in the *cis*-eQTL-rs110345285 (T/C) was associated with the up-regulation of the *eEF1A2* expression when compared with cows with the most frequent homozygous genotype (T/T) (Fig. 3h; P_FDR_ = 1.23E−10). Using the ICV-identified *cis*-eQTLs and a MAF > 10%, a significant association between the *cis*-eQTL-rs109859270 (C/T) and the absence or presence of PTB-associated lesions was observed. The heterozygous genotype in the *cis*-eQTL-rs109859270 (C/T) located in the bovine chromosome 8 was associated with higher expression levels of the U1 spliceosomal RNA (Fig. 3i; P_FDR_ = 0.0068).

The distribution of the rs43744169 and rs109859270 genotypes and odds ratio (OR) for each statistically significant genetic model and binary phenotype are presented in Table 4. For the rs43744169, the OR with 95% confidence interval (CI) under the codominant genetic model for the T/C and C/C genotypes versus the T/T genotype were 2.71 (95% CI 1.50–4.88) and 5.36 (95% CI 1.99–14.41) respectively. These results indicated that the proportion of T/C heterozygotes and C/C homozygotes with a positive ELISA, culture, and PCR result were significantly higher in the cases as compared to control cows. Under the dominant model, the genotypes T/C and C/C increased the risk of PTB compared to the genotype T/T; OR = 2.98 (95% CI 1.70–5.24). For the rs109859270 locus, the OR for the C/T and T/T genotypes versus the C/C genotype was 1.64 (95% CI 1.24–2.16), which indicated that the proportion of animals with one or two copies of the minor allele was significantly higher in animals with PTB-associated lesions as compared to control animals without lesions. Overall, the presence of the minor alleles in the rs43744169 and rs109859270 increased the PTB risk. Consequently, selecting against the rs43744169 and rs109859270 minor alleles may reduce the risk of PTB in cattle.

**Table 4:** Frequencies of the rs43744169 and rs109859270 genotypes and the odds ratio for each genetic model.

Sample	eQTL	Phenotype	Genetic model	Genotype	Phenotype frequency		OR (95% CI)
					Case (%)	Control (%)	
PB	rs43744169 (T/C)	ELISA/tissue PCR/tissue culture	Co-dominant	TT	22 (39.3)	592 (63.7)	1.00
				TC	27 (49.1)	308 (33.1)	2.71 (1.50–4.88)
				CC	6 (10.7)	31 (3.3)	5.36 (1.99–14.41)
			Dominant	TT	22 (39.3)	592 (63.7)	1.00
				TC-CC	33 (60)	339 (36.4)	2.98 (1.70–5.24)
			Log-additive	0,1,2	55(5.7)	931 (94.3)	2.45 (1.63–3.74)
ICV	rs109859270 (C/T)	Histopathology	Co-dominant	CC	279 (63.0)	367 (73.4)	1.00
				CT	148 (33.4)	119 (23.8)	1.65 (1.23–2.19)
				TT	16 (3.6)	14 (2.8)	1.54 (0.74–3.22)
			Dominant	CC	279 (63.0)	367 (73.4)	1.00
				CT-TT	164 (37.0)	133 (26.6)	1.64 (1.24–2.16)
			Recessive	CC-CT	427 (96.4)	486 (97.2)	1.00
				TT	16 (3.6)	14 (2.8)	1.32 (0.64–2.75)
			Log-additive	0,1,2	443 (47.0)	500 (53.0)	1.49 (1.17–1.89)

*PB* peripheral blood, *ICV* ileocecal valve, *OR* odds ratio, *95% CI* 95% confidence interval.

### The presence of the minor allele in the *cis*-eQTL-rs43744169 (T/C) resulted in increased *MECOM* protein levels

**Figure 4:**
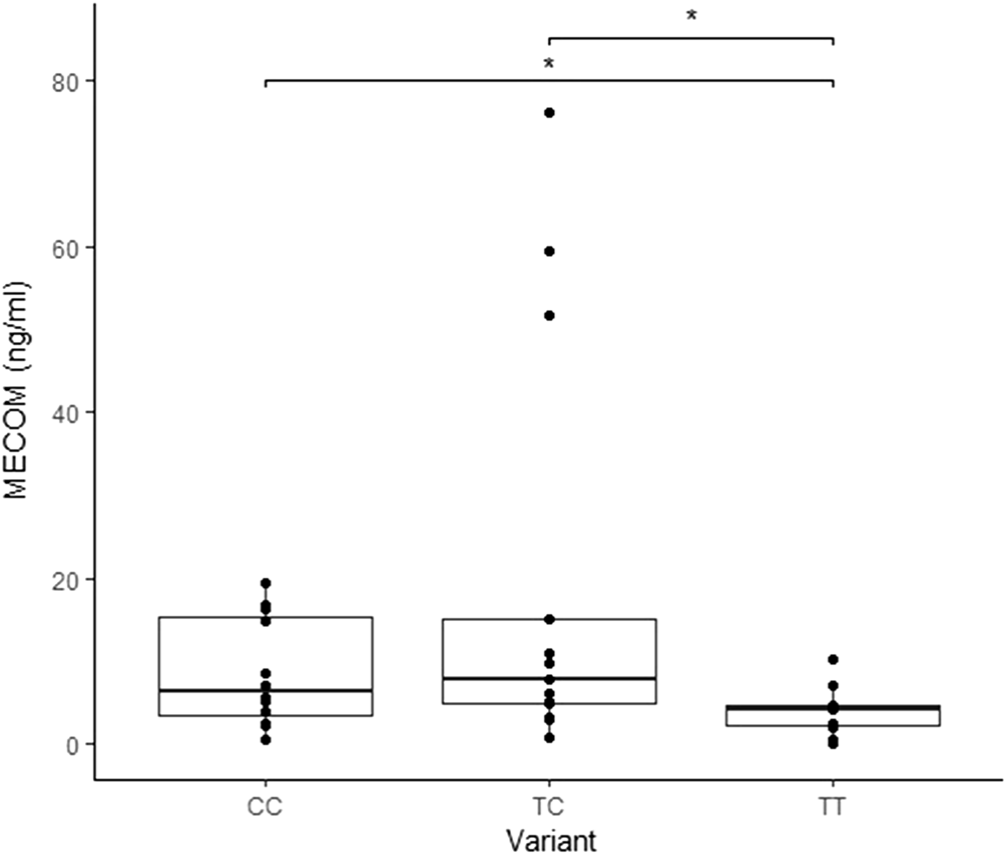
The *MECOM* expression is associated with the rs43744169. *MECOM* protein levels in plasmas from homozygous and heterozygous cows in the rs43744169 were measured with a bovine ELISA. Samples were selected from the case–control population and grouped according to the rs43744169 genotypes. Cows with the alleles T/C (N = 13) and C/C (N = 12) were associated with elevated *MECOM* protein levels when compared with the *MECOM* levels in the plasmas from cows with the two major alleles T/T (N = 11); P = 0.05 and P = 0.03, respectively. The median and the upper and lower quartiles of each group are represented by horizontal and vertical lines, respectively. The lowest point is the minimum of the data set and the highest point is the maximum of the data set. P-values were calculated using an unpaired t-test. *P-value ≤ 0.05. Each sample is represented by a circle on the dot plot.

The heterozygous genotype (T/C) in the *cis*-eQTL-rs43744169 was associated with higher *MECOM* gene expression levels (Fig. 3g, P_FDR_ = 0.00003). Using a bovine *MECOM* quantitative sandwich ELISA, we tested the *MECOM* protein levels in plasma samples from cows with focal lesions in gut tissues and with the three different rs43744169 genotypes (T/T, T/C, C/C). As seen in Fig. 4, the heterozygous (T/C) genotype and the minor allele homozygous (C/C) genotype were associated with significantly higher *MECOM* protein expression (19.60 and 8.62 ng/ml, respectively) compared with the concentration of *MECOM* in plasmas from cows with the major allele homozygous genotype (T/T, 3.90 ng/ml). This finding confirmed that increases in *MECOM* gene counts did correlate with higher levels of protein expression.

### Association between MAP survival and the *cis*-eQTL-rs43744169 genotypes using a macrophage killing assay

The association between susceptibility to MAP and the *cis*-eQTL-rs43744169 genotypes (T/T and T/C) was tested using an ex vivo monocyte-derived macrophages (MDM) killing assay. MDM were purified from peripheral blood of uninfected Holstein cattle with the *cis*-eQTL-rs43744169 genotypes T/T (N = 8) and T/C (N = 8) and infected in vitro with a virulent strain of MAP (K10 isolate). Bacterial load (log colony-forming units, log CFU) at 2 h and 7 days post-infection (p. i.) was estimated as previously described^35^. The genotype T/T showed higher MAP uptake at 2 h p.i. (P = 0.0031) and more successful MAP killing at 7 days p.i. when compared with the T/C genotype (P = 0.020) (Table 5). These results suggested a significant effect of the *cis*-eQTL-rs43744169 heterozygous genotype on bacterial survival within macrophages and confirmed that *cis*-regulatory variation modulates susceptibility to MAP infection.

**Table 5:** Genotype effect on MAP survival using a macrophage killing assay.

rs43744169	Mean log CFU-2 h p. i. (SD)	Mean log CFU-7 days p. i. (SD)	Ratio (%)	MAP survival index (SD)	P-value
T/T	4.90 (0.08)	3.51 (0.14)	71	8.46 (0.17)	0.020
T/C	4.77 (0.14)	3.53 (0.11)	74	8.60 (0.15)	

*SD* standard deviation, *CFU* colony forming units.

## Discussion

The association between expression and genotype can be tested using linear regression and ANOVA models, as well as non-linear techniques including generalized linear and mixed models, Bayesian regression, and models accounting for pedigree^30^. In the current study, we integrated gene expression data quantified by RNA-Seq and SNP data for a cohort of cows naturally exposed to MAP infection and maintained in the same environment. Using linear regression analysis, we identified 192 and 48 *cis*-eQTLs associated with the expression of 145 and 43 genes in PB and ICV, respectively. Only one of the identified cis-eQTLs, the rs109604269 (P_FDR_ = 0.011), was previously found to be associated with susceptibility to MAP infection^36^. Although the number of individual samples in this first part of the study was limited (N = 28), three of the identified *cis-* eQTLs were associated with PTB susceptibility in a larger cattle population (N = 986). Our study provides first insights into the role of *cis-*eQTLs in gene transcription regulation and PTB susceptibility. Since RNA-Seq technology is becoming increasingly affordable and provides direct information on the presence of genetic variations at the sequence level**,** further studies should be directed to call SNPs from RNA-Seq data. This approach would provide a secondary confirmation of the *cis*-eQTLs identified in the current study.

Functional analysis of the 235 genes under *cis*-regulation revealed that there was a significant enrichment of the regulation of the acute inflammatory response (GO:0002673) with three genes (*C3, S100B, SELENOS*) involved in this biological process. Heterozygosis in the *cis*-eQTLs regulating these three genes resulted in increased *C3*, *S100B,* and *SELENOS* expression. Higher *C3* levels may allow increased opsonophagocytosis and effective bacterial clearance of several microorganisms including mycobacteria^37^. *SELENOS* is involved in the degradation process of misfolded proteins in the endoplasmic reticulum, and may also have a role in inflammation control^38^. Since inflammation control depends largely on the recruitment of granulocytes (i.e., neutrophils, eosinophils, and basophils) to the inflammatory site, chemokine signaling is involved in this process. Neutrophils then secrete antibacterial proteins such as the *S100* family to destroy microbes. It is well recognized that chronic inflammatory diseases develop only when prompt and active innate immunity mechanisms, like granulocytes recruitment to the sites of infection, are ineffective or defective^39^. In fact, our recent RNA-Seq analysis from PB samples of Holstein cattle infected with MAP revealed down-regulation of the *C3, S100B, SELENOS* genes*,* and of the *CXCL8/IL8* signaling pathway, a pathway involved in neutrophils recruitment and resolution of inflammation^18^. Similarly, patients with CD have decreased secretion of *CXCL8/IL8* and reduced neutrophils migration to the sites of infection compared with healthy individuals^40^. The results presented in the current study provide evidence that genetic variants in regulatory regions (*cis*-eQTLs) can up-regulate the *C3, S100B,* and *SELENOS* gene expression which might activate a prompt resolution of the infection. However, we did not find any association between the *cis*-eQTLs regulating the *C3*, *S100B,* and *SELENOS* gene expression and PTB resistance in our case–control study. Further studies are needed to make sure that the regulation of the *C3*, *S100B,* and *SELENOS* gene expression has not a functional role in PTB resistance or tolerance.

When a functional analysis with the identified *cis*-regulated genes was performed, the cholesterol metabolic route (bta04979) was significantly enriched with the apolipoproteins *APOA4* y *APOC3* involved in this route (P_FDR_ = 0.009, Gene ratio: 2/46). The genes coding for the *APOA1* and *APOC3* are closely linked on the bovine chromosome 15 and have synergistic functions. We found that the heterozygous genotype in the rs41753850 (C/A) resulted in higher levels of *APOA4* and *APOC3* expression*,* while its absence correlated with lower levels of gene expression*.* Recent reports have demonstrated that MAP can manipulate the host lipid metabolism and accumulate cholesterol within macrophages which might favor MAP persistence^41^. Our results reinforce the notion that the lipoprotein metabolism plays an important role in PTB pathogenesis and that PTB-susceptible cows might carry alleles that increase lipoprotein production and transport. Consequently, *cis*-eQTLs associated with the *APOA4* and *APOC3* gene expression may be associated with the PTB risk. In humans, genetic variants in the *APOA1* –*APOC3–APOA4* cluster has been associated with the risk of Alzheimer disease, polycythemia induced gastric injury, and metabolic syndrome^42–44^. However, we did not find any association between the *cis*-eQTLs regulating *APOA4* and *APOC3* gene expression and PTB risk in our case–control study.

Although the number of normalized reads was low, *Matrix eQTL* found a significant association between the rs43744169 (T/C) and the *MECOM* gene expression (P_FDR_ = 0.00003). Next, we found that the presence of the minor allele in the rs43744169 (T/C) was associated with positive ELISA, PCR, and bacteriological culture results, and with increased risk of clinical PTB. Since our findings provided compelling evidence for the critical role of the *MECOM* in the progression to clinical PTB, it was prioritized in functional studies. Using a *MECOM* quantitative ELISA, we observed that the presence of the minor allele in the rs43744169 was associated with higher levels of the *MECOM* protein in plasma samples from infected cows and with increased MAP survival in an ex vivo macrophage killing assay. These findings should be confirmed in a larger population study and, additionally, by performing in vivo challenges. Allelic variants affecting the human *MECOM* gene have been associated with human inflammatory bowel disease^45^. Mutations resulting in aberrant expression of the *MECOM* have also been found in many types of solid cancers, including colorectal cancer, as well as in acute myeloid leukemia^46,47^. Previous scientific evidence showed that the *MECOM* acts as an oncogene regulating signaling pathways that lead to increased tumor proliferation^48^. More specifically, cell line assays have demonstrated a role for the *MECOM* in the transcriptional control of *c-fos*, transforming growth factor* β* (*TGF*-*β*) and the *activator protein 1* (*AP1*) proliferative pathway^49^. The *MECOM* also binds to the promoter regions of the majority of genes involved in the *JAK-STAT* signaling pathway^50^. Excessive *JAK-STAT* signaling activation results in numerous inflammatory and hematopoietic disorders. Recent findings indicate that the *MECOM* is up-regulated by inflammatory stimuli, including bacteria, and that mutations in the *MECOM* made mice more susceptible to bacterial infections^51^. The *MECOM* has also a recognized function as a crucial regulator of the nuclear factor-kappa β (*NF-κβ*)-mediated inflammatory response^52^. *NF-κβ* is a critical factor in the gut immune responses to pathogens and in promoting inflammation-associated carcinoma in the gastrointestinal tract^53^. Our findings suggest that genetic variants in a *cis*-eQTL affecting the expression of the *MECOM,* a transcriptional regulator of the *NF-κβ* inflammatory response*,* may cause an uncontrolled and aberrant inflammatory response which might exacerbate tissue injury in PTB-infected cattle. This is in agreement with Kiser et al., who found an association between seven genes linked to the *NF-κβ* pathway (*DUSP10, IKBKB, NR4A1, PRKCA, SLC2A5, TGFB2,* and *PIK3R1*) and MAP tissue infection in two Holstein populations using gene set enrichment analysis-SNP (GSEA-SNP)^16^.

Our case–control association study revealed a second *cis*-eQTL, the rs110345285 (T/C), associated with the up-regulation of the *eEF1A2* gene expression and with high levels of MAP antibodies. More specifically, increased ELISA (OD) values were associated with the less frequent homozygous genotype (C/C) in the *cis*-eQTL-rs110345285. The *eEF1A2* is a protein translation factor involved in protein synthesis and with important anti-apoptotic, migration and pro-metastasis functions on cancer development^54,55^. Mechanistic studies revealed that the *PI3K/Akt* /*NF-κβ* and *JAK/STAT* signaling pathways play an important role in mediating the effects of the *eEF1A2*^56^. *eEF1A2* expression increases the formation of filopodia structures that have an important role in driving cell migration and invasion^57^. High *eEF1A2* expression is associated with severe tumor grades and metastasis in several cancer cell lines and with some cases of multiple myeloma, a plasma cell neoplasm of humans^58^. Furthermore, the *eEF1A2* blocks apoptosis and favors viral replication^59^. In agreement with this, our results suggested that PTB-infected cows carrying the risk allele in the rs110345285 (C) and exhibiting high levels of *eEF1A2* might have a reduced probability of survival compared to their non-*eEF1A2*-expressing counterparts.

Our case–control association analysis revealed a third significant association between the *cis*-eQTL-rs109859270 (C/T) and the absence or presence of PTB-histopathological lesions. For the rs109859270 locus, the OR for the C/T and T/T genotypes versus the C/C genotype was 1.64 (95% CI 1.24–2.16), which indicated that the proportion of animals with one or two copies of the minor allele were significantly higher in animals with PTB-associated lesions as compared to control animals without lesions. In addition, the heterozygous genotype in the *cis*-eQTL-rs109859270 was associated with the up-regulation of the U1 spliceosomal RNA expression. Splicing is performed by a spliceosome that assembles on each intron and predominantly comprises U1, U2, U4, and U5, and U6 uridyl-rich small nuclear RNPs (snRNPs) in equal stoichiometry^60,61^. U1 snRNPs plays an essential role in defining the 5′ end splice site by RNA-RNA base pairing. Besides its splicing role, U1 snRNPs protects pre-mRNAs from drastic premature termination by premature cleavage and polyadenylation (PCPA) at more proximal alternative polyadenylation sites, which has already been proved in activated immune, neuronal, and cancer cells^62^. U1 snRNA abnormalities can cause defects in pre-mRNA splicing, which are considered as a primary cause of several diseases^63^. In other words, mammalian cells require U1 snRNA with expression levels in a restricted range to prevent drastic premature termination of the nascent pol II transcripts by PCPA^64^. In human macrophages, U1 snRNA over-expression has been associated with impairment of autophagosome-lysosome fusion^65^. Recent studies reported that the infection of human macrophages with *Mycobacterium tuberculosis* (MTb) results in massive alterations in the pattern of RNA splicing in the host^66,67^. Furthermore, two genes related to macrophage function, the monocyte to macrophage differentiation gene (*MDM*) and adenosine deaminase (*ADA*) genes, were differentially spliced in MAP-infected ileum suggesting a possible mechanism by which MAP escapes the host innate immune response^68^. Our findings suggested that the heterozygous genotype in the *cis*-eQTL-rs109859270 (C/T) resulted in increased U1 spliceosomal RNA expression levels. In contrast, the most frequent homozygous genotype (C/C) maintained the U1 snRNA expression levels in a restricted range and was associated with a more controlled risk of infection. Further studies are required to understand the mechanisms of splicing and alternative splicing regulation upon MAP infection and how they can impact immune responses in cattle.

## Conclusions

The identification of functional variants (*cis-eQTLs*) followed by a case–control association analysis allowed the identification of three *cis*-eQTLs regulating the expression of the *MECOM,* e*EF1A2,* and U1 spliceosomal RNA expression and their validation as significantly associated with PTB susceptibility in a larger cattle population. It should be emphasized that *MECOM*, *eEF1A2,* and U1 risk alleles have shown detrimental effects in other inflammatory diseases including human IBD and colorectal cancer. Furthermore, the introduction of the cis-eQTLs described in the current study into marker-assisted breeding programs might reduce the disease through selection, reduce economic losses, and improve animal health.

## Materials and methods

### Ethics statement

Experimental procedures were approved by the Animal Ethics Committee of the Servicio Regional de Investigation y Desarrollo Agroalimentario (SERIDA) and authorized by the Regional Consejeria de Agroganadería y Recursos Autoctonos of the Principality of Asturias (authorization code PROAE 29/2015). All the procedures were conducted following 2012/63/EU of the European Parliament. Blood, fecal, and tissue samples were collected by trained personnel and following good veterinary practice. Animals used in the case–control study were sampled under pertinent legislation for safeguarding animal welfare (Basque Government Decree 454/1994, Spanish Government Law 32/2007, Royal decree 731/2007, and European council regulation number 1099/2009).

### Animals and PTB diagnosis

PB and ICV samples were collected from fourteen Holstein Friesian cows from a single commercial dairy farm in Asturias (Spain) at the time of slaughter. This farm was selected for sample collection based on PTB clinical cases, with some cows confirmed by necropsy examination. The mean prevalence of the disease in the farm (period 2016 to 2019) was estimated at 6.29% by ELISA for the detection of MAP antibodies. Infection status was determined by histopathological analysis of gut tissues, ELISA, bacteriological culture, and PCR assays as previously described^28^. For the case–control study, the infection status of 986 Holstein Friesian cattle from eight Northeast Spain regions was determined by histopathological analysis of gut tissues, ELISA for MAP antibodies detection, and tissue culture and PCR for MAP detection^32^. Animals were 5.5 years old on average. Except for six animals that were aged between 18 and 23.2 months, the majority of the animals were adults (2 years or older).

### Genotyping

Blood samples were collected from the coccygeal (tail) vein into EDTA Vacutainer tubes (BD Vacutainer System, Becton, Dickinson, and Company, Sparks, MD, USA). Blood samples were centrifuged at 2500×*g* for 20 min at 4 °C and buffy layers containing white blood cells were collected. Genomic DNA was extracted from the blood buffy coat using the QIAmp DNA Blood Mini Kit according to the manufacturer’s instructions (Qiagen, Hilden, Germany). Purified genomic DNA was quantified spectrophotometrically and subsequently genotyped on the EuroG10K BeadChip at the molecular genetic laboratory service of the Spanish Federation of Holstein Cattle (CONAFE) using the *Infinium iScan* software for allele assignation (Illumina, San Diego, CA) as previously described^28^. The EuroG10K BeadChip is a development by EuroGenomics and its collaborators.

### Gene expression data

In the current study, the alignment files from our previous RNA-Seq study were used^18^. Briefly, PB samples were collected by venipuncture of the coccygeal vein using PAXgene Blood RNA tubes (2.5 ml) (Qiagen, Hilden, Germany). Total RNA was purified from the PB samples using the PAXgene blood RNA kit according to the manufacturer´s instructions (Qiagen, Hilden, Germany). For RNA isolation, 150–200 mg of ICV were harvested and immediately submerged in 2 ml of RNA later (Sigma, St. Louis, MO). Purification of RNA was performed using the RNeasy Mini Kit according to the manufacturer’s instructions (Qiagen, Hilden, Germany). Residual genomic DNA was removed using DNase digestion with RNase-free DNase I amplification grade following the recommended protocol (Invitrogen, Spain). The concentration and quality of the total RNAs were measured using an Agilent Bioanalyzer 2100 (Agilent Technologies, Santa Clara, CA, US). All samples had an RNA integrity value of 7 or greater. Approximately 250 ng of RNA were used for RNA-Seq library preparation using the Illumina NEBNext Ultra Directional RNA Library preparation kit following the manufacturer´s instructions (Illumina, San Diego, CA, US). Libraries quality was assessed using an Agilent Bioanalyzer and Agilent high sensitivity DNA chip to confirm that the insert sizes were 188–274 bp for all the individual libraries. RNA-Seq libraries were single-end sequenced in a 1 × 75 bp format using an Illumina NextSeq 500 sequencer at the Genomic Unit of the Scientific Park of Madrid, Spain. All the libraries were sequenced generating an average of 22.31 million raw reads per library with a Phred quality score > 30. The raw reads were filtered by their length (minimum size 75 bp long) and percentage of ambiguous base N less than 10% using *Prinseq-lite 0.20.4*^25^. After quality control, reads were subsequently mapped to the *Bos Taurus* reference genome (Bos_taurus.UMD3.1. version 87) with *TopHat 2.1.1*^26^. The resulting alignment files (.bam) were provided to the *Cufflinks* package *2.2.1*^69^ to calculate gene expression levels and genes differentially expressed in the infected animals with focal/multifocal or diffuse lesions versus the control group.

In the current study, the alignment files (.bam) from our previous RNA-Seq study^18^ were used to generate a table of reads for each sample using *Rsubread*^27^. The number of reads mapped to 24,616 genes was counted using the function feature counts in the *Rsubread* package. Next, the analysis of differential expression between the cows with focal/multifocal or diffuse lesions versus control cows was conducted using *DESeq2*^29^. The differentially expressed genes were selected as those with an adjusted P- value (P_FDR_) ≤ 0.05.

### *cis*-eQTL analysis

Gene expression data (read counts) were normalized with the mean-of-ratios method included in the *DESeq2* package^29^. Then, *R Matrix eQTL 2.2* was used to test for associations between each SNP-normalized counts by modeling the effect of genotype as additive linear (least square model), where the null hypothesis is no association between genotype and gene expression. This tool implements matrix covariance calculation and efficiently runs linear regression analysis for *cis*-eQTL discovery^30^. *cis*-eQTLs were calculated by selecting gene variants on the same chromosome that are located within 1 Mb upstream of the TSS of a gene locus. Specific details regarding the matrix structure in *Matrix eQTL* have been previously described^70^. Briefly, S and G denote the genotype matrix and the gene expression matrix, respectively. Each row of these matrices holds different measurements for a single SNP among samples and a single gene across samples, respectively. Data matrices are sliced into blocks of up 10,000 variables. Then, gene expression and genotype matrices are standardized. For each pair of blocks, the correlation matrix for a relevant block is calculated and *Matrix eQTL* checks if the absolute value of any correlation exceeds a predefined threshold. Then, *Matrix eQTL* calculates the P-values only for those SNP-normalized counts associations selected based on their correlations being larger than the threshold. *Matrix eQTL* uses the false discovery rate (FDR)^71^ to adjust for multiple hypotheses testing and to identify significant *cis*-eQTLs (P_FDR_ ≤ 0.05). FDR is the expected proportion of false positives among all the significant tests.

### Functional enrichment analysis

Genes with significant *cis*-eQTLs were investigated for enrichment of biological processes using gene ontology categories^72^ within the *cluster Profiler Bioconductor package 3.10.1*^31^ and Panther database^73^. A threshold for significant enrichment was set at P_FDR_ ≤ 0.05 after adjustment with the Benjamini–Hochberg procedure^71^.

### Case–control study

Associations between genotypes-phenotypes were assessed by logistic regression analysis using the WGassociation function of *SNPassoc 1.9.2* under five different genetic models (co-dominant, dominant, recessive, over-dominant, and log-additive)^34^. The response variable can be binary (positive or negative diagnostic result) or continuous (ELISA OD values and PTB-associated lesions classified according to their severity in five grades). The genotypes of the *cis-* eQTLs were included as possible explanatory variables. The WGassociation function fits individual logistic regression models to each of the class variables (genotypes) using age as a covariate. P-values were adjusted using the Bonferroni correction (based on the total number of markers tested) for multiple comparisons. For each *cis*-eQTL and genetic model, the function WGstats of *SNPassoc 1.9.2.* provided genotype frequencies, OR (or mean differences for quantitative traits), and 95% CI with the major homozygous genotype deemed as the baseline.

### Bovine *MECOM* ELISA kit

The levels of *MECOM* protein in plasma samples (50 µl) were assessed by using a quantitative sandwich ELISA according to the manufacturer’s instructions (MyBioSource, San Diego, US). The sensitivity of the kit is 1.0 ng/ml and the detection range is 3.12–100 ng/ml. Briefly, standards and samples (50 µl) were added in duplicate into an appropriate ELISA plate. One hundred microliters of the horseradish peroxidase-conjugated antibody was added to each well. After incubation for 60 min at 37 °C, the plate was washed four times with 350 µl of wash solution and incubated with 50 µl of 3, 3′, 5, 5′-Tetramethylbenzidine for 15 min at 37 °C in the dark. After adding 50 µl of stop solution into each well, the OD values were measured in an ELISA reader at 450 nm (Thermo Scientific Multiskan, US). We average the duplicate readings for each standard and sample to subtract the average OD of the blank. A standard curve was generated by plotting the mean OD values of each standard on the vertical axis and the corresponding concentration on the horizontal axis. The concentration level of the *MECOM* in each sample was interpolated from the standard curve. Statistical analysis was performed using an unpaired t-test for comparison between two groups (*GraphPad Prism 8*, San Diego, California, US). Differences were considered significant when P ≤ 0.05.

### Macrophage killing assay

Peripheral blood was drawn from healthy Holstein cows into heparinized Vacutainer tubes (Becton, Dickinson and Company, Sparks, MD), diluted 1:2 in Hanks balanced salt solution (HBSS), layered over 10 ml of Ficoll-Paque gradient (1.084 g/cm^3^) (GE HelthCare, Uppsala, Sweden) and centrifuged at 900×*g* for 30 min. The cell interphase was collected and centrifuged at 400×*g* for 10 min to remove platelets from peripheral blood mononuclear cells (PBMC). PBMC were resuspended in RPMI-1640 supplemented with 20 mM l-glutamine, 10% heat-inactivated bovine serum, 100 U/ml penicillin G, and 100 mg/ml streptomycin sulfate (Lonza, Spain) and seeded at a density of 1 × 10^6^ PBMC/ml into 24-well tissue culture plates. After 2 h at 37 °C in a humidified 5% CO_2_ incubator, non-adherent cells were removed. Adherent cells were incubated for 7 days at 37 °C to allow differentiation to MDM before infection. Differentiated MDM were inoculated in triplicate with a single-cell suspension of MAP reference strain K10 at a multiplicity of infection (bacteria:cells) of 10:1. After 2 h, the supernatant was removed, and the cells were washed twice with HBSS to remove extracellular bacteria. Infected MDM were lysed at this time (2 h p.i.) or cultured at 37 °C for 7 days in fresh medium. At each time point, the supernatant was aspirated and infected MDM were lysed by vigorous pipetting with 0.5 ml of 0.1% Triton X-100 (Sigma-Aldrich) in sterile water for 10 min. Supplemented Mycobacteria Growth indicator tubes (MGIT) (Becton, Dickinson and Company, Sparks, MD, US) were inoculated with 0.1 ml of each initial bacterial suspension and with the 2 h and 7 d p. i. cell lysates. The tubes were incubated at 37 ± 2 °C for up to 41 days in a Bactec MGIT 960 instrument (Becton, Dickinson and Company). The earliest instrumental indication of positivity (i.e., time to detection [TTD]) for each tube was recorded. The predicted number of bacteria in each positive tube was calculated using previously generated mathematical equations that relate TTD (in days) to the estimated log CFUs^35^. The CFU ratios between 2 h and 7 days p.i. were calculated by dividing the estimated log CFUs at day 7 by that at 2 h p.i. MAP survival index was estimated as the square root CFU ratio × 100. Higher values of the MAP survival index reflect higher susceptibility to infection. A student’s *t*-test was performed for comparison of the survival indexes between two groups (*GraphPad Prism 8*, San Diego, CA, US). Differences were considered significant when P-values were ≤ 0.05.

## Supplementary Information

Supplementary Table S1.

Supplementary Table S2.

Supplementary Table S3.

## Data Availability

RNA-Seq raw data have been deposited in the NCBI Gene Expression Omnibus (GEO) database under the accession number GSE137395. SNPs datasets will not be made publically available as the data were generated from private-owned cattle, with a legal commitment to confidentiality. Researchers may request access to the data and consideration will be given to individuals following the conclusion of a confidentiality agreement.
