# Author Correction: Identification of loci associated with susceptibility to bovine paratuberculosis and with the dysregulation of the *MECOM*, *eEF1A2*, and U1 spliceosomal RNA expression

**DOI:** 10.1038/s41598-021-00840-3

**Published:** 2021-10-25

**Authors:** Maria Canive, Nora Fernandez‑Jimenez, Rosa Casais, Patricia Vázquez, José Luis Lavín, José Ramón Bilbao, Cristina Blanco‑Vázquez, Joseba M. Garrido, Ramón A. Juste, Marta Alonso‑Hearn

**Affiliations:** 1 Department of Animal Health, NEIKER- Basque Research and Technology Alliance (BRTA), Derio, Bizkaia Spain; 2 Doctoral Program in Immunology, Microbiology and Parasitology, Universidad del País Vasco/Euskal Herriko Unibertsitatea (UPV/EHU), Leioa, Bizkaia Spain; 3 Department of Genetics, Physical Anthropology and Animal Physiology, University of the Basque Country (UPV/EHU), Biocruces-Bizkaia HRI, Leioa, Bizkaia Spain; 4 SERIDA, Servicio Regional de Investigación y Desarrollo Agroalimentario, Grupo NySA, Deva, Asturias Spain; 5 SALUVET‑Innova S.L., Faculty of Veterinary Sciences, Complutense University of Madrid, Madrid, Spain; 6 Department of Applied Mathematics, NEIKER-Basque Research and Technology Alliance (BRTA), Derio, Bizkaia Spain

Correction to: *Scientific Reports* 10.1038/s41598-020-79619-x, published online 11 January 2021

The original version of this Article omitted an affiliation for Maria Canive. The correct affiliations are listed below.

Department of Animal Health, NEIKER- Basque Research and Technology Alliance (BRTA), Derio, Bizkaia, Spain.

Doctoral Program in Immunology, Microbiology and Parasitology, Universidad del País Vasco/Euskal Herriko Unibertsitatea (UPV/EHU), Leioa, Bizkaia, Spain.

The original Article and accompanying Supplementary Information files have been corrected.

